# Dorso-ventral heterogeneity in tracheal basal stem cells

**DOI:** 10.1242/bio.058676

**Published:** 2021-09-14

**Authors:** Tomomi Tadokoro, Keisuke Tanaka, Shun Osakabe, Mimoko Kato, Hisato Kobayashi, Brigid L. M. Hogan, Hideki Taniguchi

**Affiliations:** 1 Department of Regenerative Medicine, Yokohama City University Graduate School of Medicine, Yokohama, Kanagawa 236-0004, Japan; 2 Department of Cell Biology, Duke University School of Medicine, Durham, NC 27707, USA; 3 Division of Regenerative Medicine, Center for Stem Cell Biology and Regenerative Medicine, Institute of Medical Science, University of Tokyo, Minato, Tokyo 108-8639, Japan; 4 NODAI Genome Research Center, Tokyo University of Agriculture, Setagaya, Tokyo 156-8502, Japan; 5 Department of Embryology, Nara Medical University, Kashihara, Nara 634-8521, Japan

**Keywords:** Basal stem cells, Tracheal epithelium, Dorso-ventral difference, Basal cell heterogeneity

## Abstract

The tracheal basal cells (BCs) function as stem cells to maintain the epithelium in steady state and repair it after injury. The airway is surrounded by cartilage ventrolaterally and smooth muscle dorsally. Lineage tracing using Krt5-CreER shows dorsal BCs produce more, larger, clones than ventral BCs. Large clones were found between cartilage and smooth muscle where subpopulation of dorsal BCs exists. Three-dimensional organoid culture of BCs demonstrated that dorsal BCs show higher colony forming efficacy to ventral BCs. Gene ontology analysis revealed that genes expressed in dorsal BCs are enriched in wound healing while ventral BCs are enriched in response to external stimulus and immune response. Significantly, ventral BCs express Myostatin, which inhibits the growth of smooth muscle cells, and HGF, which facilitates cartilage repair. The results support the hypothesis that BCs from the dorso-ventral airways have intrinsic molecular and behavioural differences relevant to their *in vivo* function.

## Abstract (precis)

**Summary:** Spatial difference of tracheal epithelium, especially focused on the heterogeneity of basal stem cells, is elucidated by lineage tracing *in vivo*, histological analysis, tracheosphere culture, and gene ontology analysis.

## INTRODUCTION

The mouse trachea serves as a convenient and well-established model system for the conducting airways of the human lung (Rock et al., 2010). It is lined by a pseudostratified mucociliary epithelium primarily composed of ciliated and secretory luminal cells, and is maintained and repaired by an underlying population of basal cells (BCs) (Rock et al., 2009). The trachea is surrounded by cartilaginous rings on the ventral and lateral sides, and smooth muscle on the dorsal side, and is supplied with blood vessels and nerves. The upper part of the mouse trachea between the cartilaginous rings also contains submucosal glands (SMGs), which are known to serve as facultative stem cell niches. Myoepithelial cells in the SMGs can repair the airway epithelium after severe injury (Hegab et al., 2011; Tata et al., 2018; Lynch et al., 2018).

During mouse development, the tracheal primordium was observed to form at E9.5 and continued to be elongated throughout the tracheal development period. Cartilage development was observed to commence at E10.5, characterised by the expression of SOX9-positive cells, whereas smooth muscle development commenced at E11.5, characterised by the expression of ACTA2 (Hines et al., 2013). Cartilage segmentation begins from E13.5 (Young et al., 2020). With respect to the development of airway epithelium, TRP63-positive BCs and SCGB3A2-positive secretory cells were observed in NKX2-1-positive airway epithelium from E10.5 (Kurotani et al., 2008; Hou et al., 2019), whereas FOXJ1-positive ciliated cells were observed to form from E14.5 (Tsao et al., 2009). There is increasing evidence that supports the existence of reciprocal epithelial-mesenchymal interactions in the development of the mouse trachea. For example, a recent study demonstrated that the mesenchyme regulates elongation along the anterior-posterior axis and the expansion of the developing trachea (Kishimoto et al., 2018; Young et al., 2020). Wnt5a-Ror2 signalling in mesenchymal cells regulates the elongation of the trachea through the polarisation of smooth muscle progenitors. Genetic disruption of cartilage or smooth muscle formation affects BC composition and differentiation in tracheal epithelium, which indicates the importance of the mesenchyme for the normal development of the tracheal epithelium (Hines et al., 2013). In addition, a recent study has revealed that the loss of Wnt secretion in epithelial cells affects cartilage formation from mesenchymal cells and results in the loss of FGF10 which affects the differentiation of airway epithelial cells, including BCs (Hou et al., 2019).

The varying distribution of BC subpopulations in the ventral and dorsal trachea was observed in the developmental stage, whereas this was not observed in the adult stage (Hines et al., 2013; Hou et al., 2019). However, clear differences were observed in the mucocilliary transport of particles in the airway epithelium on the ventral and dorsal surfaces of the trachea (Hoegger et al., 2014); the particles were transported preferentially along the ventral trachea, implicating the dorso-ventral difference in the cellular composition of the adult tracheal epithelium. Recent studies using single-cell RNA sequencing (scRNA-seq) analysis revealed the existence of different types of BCs (Watson et al., 2015; Montoro et al., 2018; Braga et al., 2019; Travaglini et al., 2020); however, their roles and spatial distribution pattern are yet to be fully elucidated.

In this study, we demonstrate the dorso-ventral variance in the intrinsic behaviour of BCs in the adult airway epithelium and suggest the potential role of tracheal BCs in homoeostasis in the tracheal mesenchyme.

## RESULTS

### Dorso-ventral difference in cell expansion from tracheal basal stem cells

**Fig. 1.**
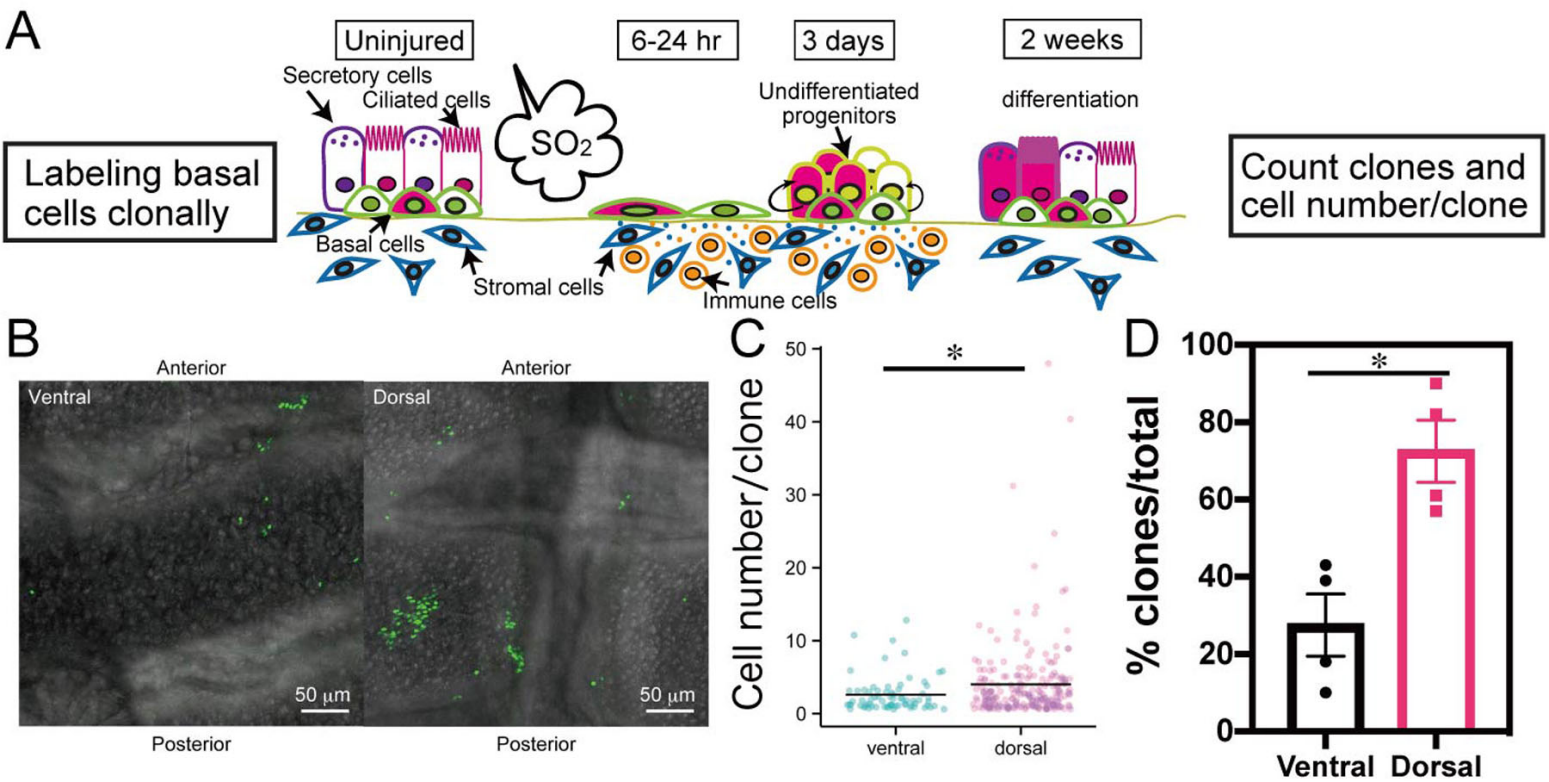
***In vivo* clonal expansion of basal stem cells during repair.** (A) Schematic representation of clonal analysis using *Krt5-Cre^ERT2^; Rosa-Confetti* mice. Single cells treated with a low dose of tamoxifen (magenta) exhibited proliferation after SO_2_-induced injury and differentiated into ciliated and secretory cells. (B) Confocal images of clones expressing nuclear GFP (green) in ventral and dorsal trachea specimens 2 weeks after SO_2_-induced injury. (C) Jitter plot of cell number per clone in ventral and dorsal trachea (*n*=71 clones for ventral trachea and *n*=224 clones for dorsal trachea). (D) Dorso-ventral distribution of basal cell (BC)-derived clones (*n*=4 tracheas each). Scale bars: 50 µm. Data are represented as mean±s.e.m. *P<0.05. See also Fig. S1.

Brief treatment of mice with sulphur dioxide gas has been observed to cause the death of luminal cells in the trachea, and was closely followed by the proliferation of surviving BCs and their differentiation into ciliated and secretory cells (Rock et al., 2009). To determine whether there is heterogeneity in the reparative behaviour of BCs in the dorsal versus ventral trachea, we performed clonal lineage tracing of *KRT5-CreER^T2^*; *Rosa-Confetti* mice (Fig. 1A). Two weeks after the treatment, we determined the number and sizes of individual clones of cells labelled with a nuclear green fluorescent protein (GFP) reporter on the dorsal half versus the ventral half of the trachea (Fig. 1B). In addition to the airway epithelial cells, a few myoepithelial cells were lineage-labelled after 1 year without injury (Fig. S1A), suggesting that some of the clones in the ventral trachea could be derived from myoepithelial cells present in the SMGs. The results showed that, on an average, the clones in the dorsal domain (average of 4.03 cells/clone) were larger than those in the ventral domain (average of 2.61 cells/clone, Fig. 1C). The proportion of clones in the dorsal trachea was also higher than that in the ventral trachea (72% and 28%, respectively, Fig. 1D). Long-term lineage tracing of BCs also showed that the large clones were located in the dorsal trachea between the cartilage and smooth muscles (Fig. S1B). These results suggest that there are intrinsic differences in the stem cell behaviour of dorsal versus ventral BCs, or differences in the stem cell environment in the two domains.

### Dorso-ventral differences in tracheal luminal cells, vascular networks, neurons, and the expression of BC markers

**Fig. 2.**
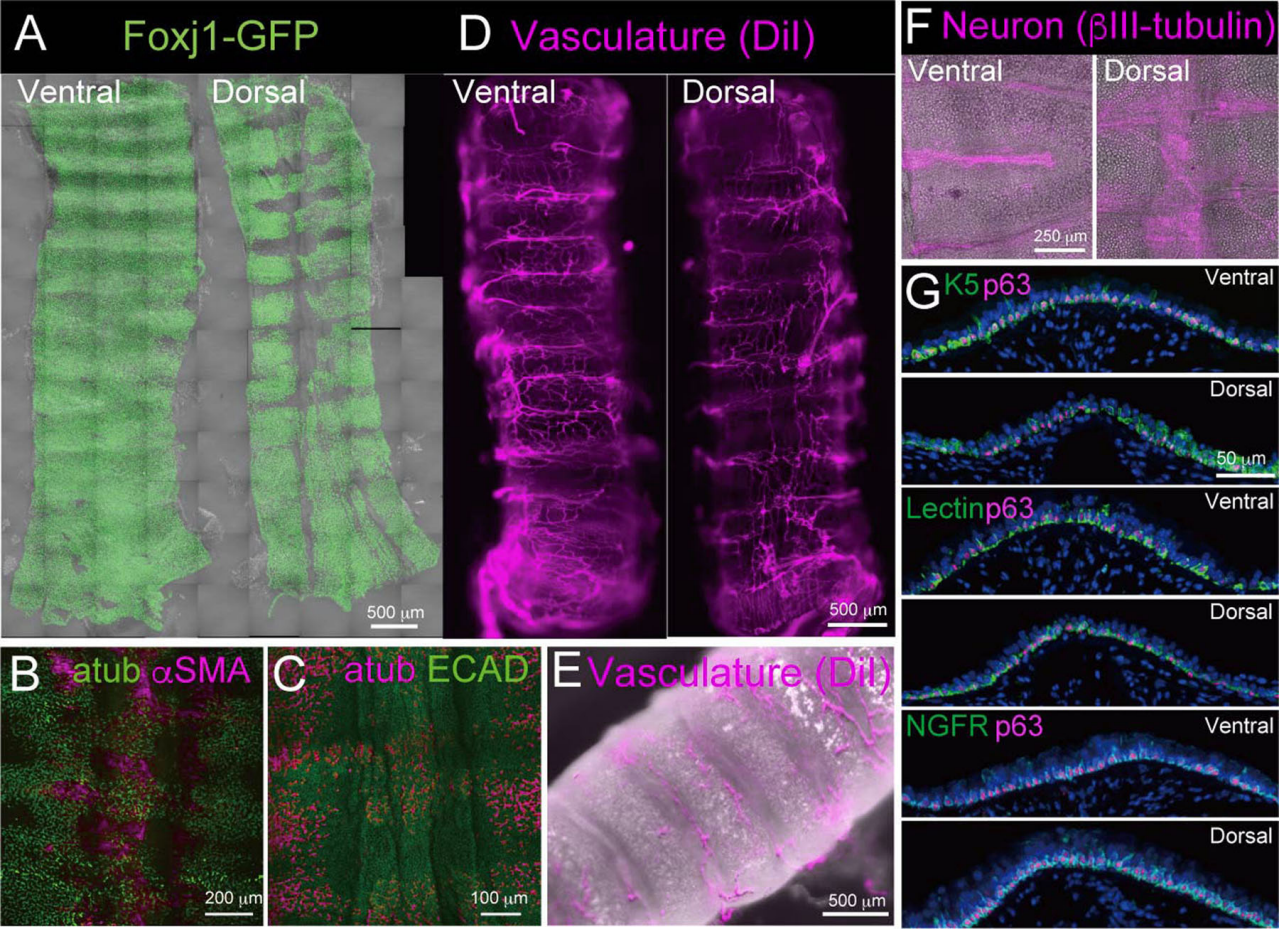
**Comparison of airway epithelium, vascular networks, nerve fibers and BC marker expression in ventral and dorsal trachea specimens.** (A) Whole-mount imaging of ciliated cells (green; *Foxj1*-GFP) in mouse trachea. (B) Whole-mount imaging of ciliated cells (green; acetylated tubulin) and smooth muscle cells (magenta; αSMA) in dorsal trachea. (C) Whole-mount imaging of ciliated cells (green; atub) and epithelial cells (magenta; ECAD) in dorsal trachea. (D) Whole mount imaging of blood vessels by cardiac perfusion of DiI (magenta). (E) Stereomicroscopical image of trachea (Bright field) and blood vessels (magenta; Dil). (F) Whole-mount images of nerve fibers (magenta; βIII-tubulin) from ventral and dorsal trachea. (G) Immunofluorescence staining of multiple BC markers (green; KRT5, GSI-B4, and NGFR; magenta; p63). See also Table S1.

To identify any differences in the spatial distribution of cell types in the dorsal versus ventral trachea, we compared the confocal images of epithelial cells, smooth muscles and neurons in whole-mount samples and the stereomicroscopic images of vascular networks and performed immunohistochemical analysis of BCs in tissue sections (Fig. 2A-G). In the ventral region, ciliated cells were distributed uniformly, including above and between the cartilaginous rings. However, in the dorsal region, there were fewer ciliated cells present above the smooth muscles while that area was covered by epithelial cells (Fig. 2A-C). Next, we studied the vascular networks in the whole trachea by cardiac perfusion of the fluorescent dye DiI (Fig. 2D,E). The images show that blood vessels span the spaces between the cartilages, and several vessels span vertically to connect the cartilages (Fig. 2D,E). The blood vessels were marginally more abundant in the ventral trachea than in the dorsal trachea (Fig. 2D). Similarly, neurons were mainly distributed between the cartilages in both ventral and dorsal trachea (Fig. 2F). In addition, neurons were found in the smooth muscle area (Fig. 2F). Lastly, the expression of various BC markers (KRT5, GSI-B4 lectin, and NGFR co-stained with TRP63) was examined in both ventral and dorsal tracheal specimens (Rock et al., 2009) (Fig. 2G). Almost 84% of GSI-B4 lectin positive cells and 89% of KRT5 positive cells showed co-staining for TRP63, a marker of BCs derived from tracheal, oesophageal, and skin epithelium (Pellegrini et al., 2001; Daniely et al., 2004), and there was almost no difference in the ratio of the double-positive cell population between the ventral and dorsal trachea (84.38±1.72% versus 84.88±1.77% and 88.57±1.08% versus 89.35±1.31%, respectively). Conversely, the proportion of NGFR/TRP63 double-positive cell population differed significantly between the ventral and dorsal trachea (85.16±2.04% and 92.12±1.2%, respectively; *P*<0.01), suggesting the difference in the expression of BC markers (Fig. 2G and Table S1).

### Comparison of BCs from ventral and dorsal trachea using *in vitro* tracheosphere culture

**Fig. 3.**
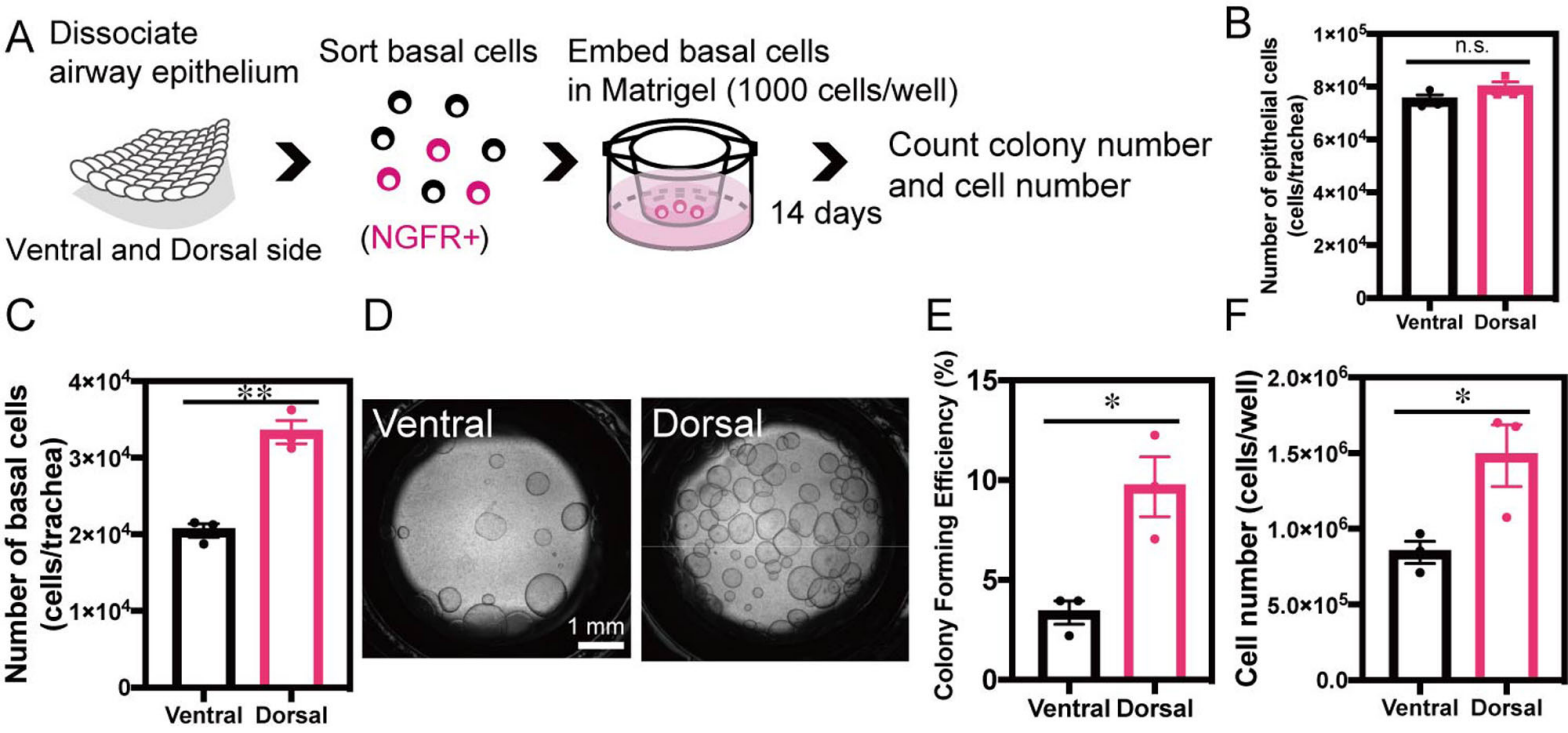
***In vitro* tracheosphere formation from BCs derived from ventral and dorsal trachea.** (A) Schematic representation of BC isolation from mouse trachea and 3D tracheosphere culture. (B) Number of epithelial cells isolated from ventral and dorsal trachea (*n*=3). (C) Number of BCs isolated from ventral and dorsal trachea (*n*=3). (D) Phase-contrast images of colonies formed after 2 weeks of culture. (E) Colony-forming efficiencies of BCs derived from ventral and dorsal trachea (*n*=3). (F) Cell numbers after 2 weeks of culture of BCs derived from ventral and dorsal trachea (*n*=3). Data are represented as mean±s.e.m. *P<0.05, **P<0.01.

Next, we conducted a tracheosphere assay to estimate the intrinsic ability of BCs to proliferate and differentiate in a culture medium without stromal cells. The trachea specimens were separated into dorsal and ventral sections, and the NGFR-positive BCs were sorted separately after dissociation from the epithelium (Fig. 3A). One thousand cells per well were cultured in Matrigel for 14 days, and the number of colonies and cells was determined (Fig. 3A). Cell-sorting experiments showed that NGFR-positive BCs were more abundant in the dorsal trachea than in the ventral trachea, while cell number of total epithelia was not changed in the two groups (Fig. 3B,C). Although the same number of BCs were seeded, the colony-forming efficiencies differed between the tracheosphere cultures of the ventral and dorsal trachea specimens (3.4% and 9.7%, respectively) (Fig. 3D,E). The number of cells was also higher in the tracheospheres derived from dorsal BCs (Fig. 3F). These results suggest that the difference in cell expansion observed both *in vitro* and *in vivo* may be attributed to the differences in the characteristics of BCs derived from the ventral and dorsal trachea.

### Gene expression analysis of BCs derived from ventral and dorsal trachea

**Fig. 4.**
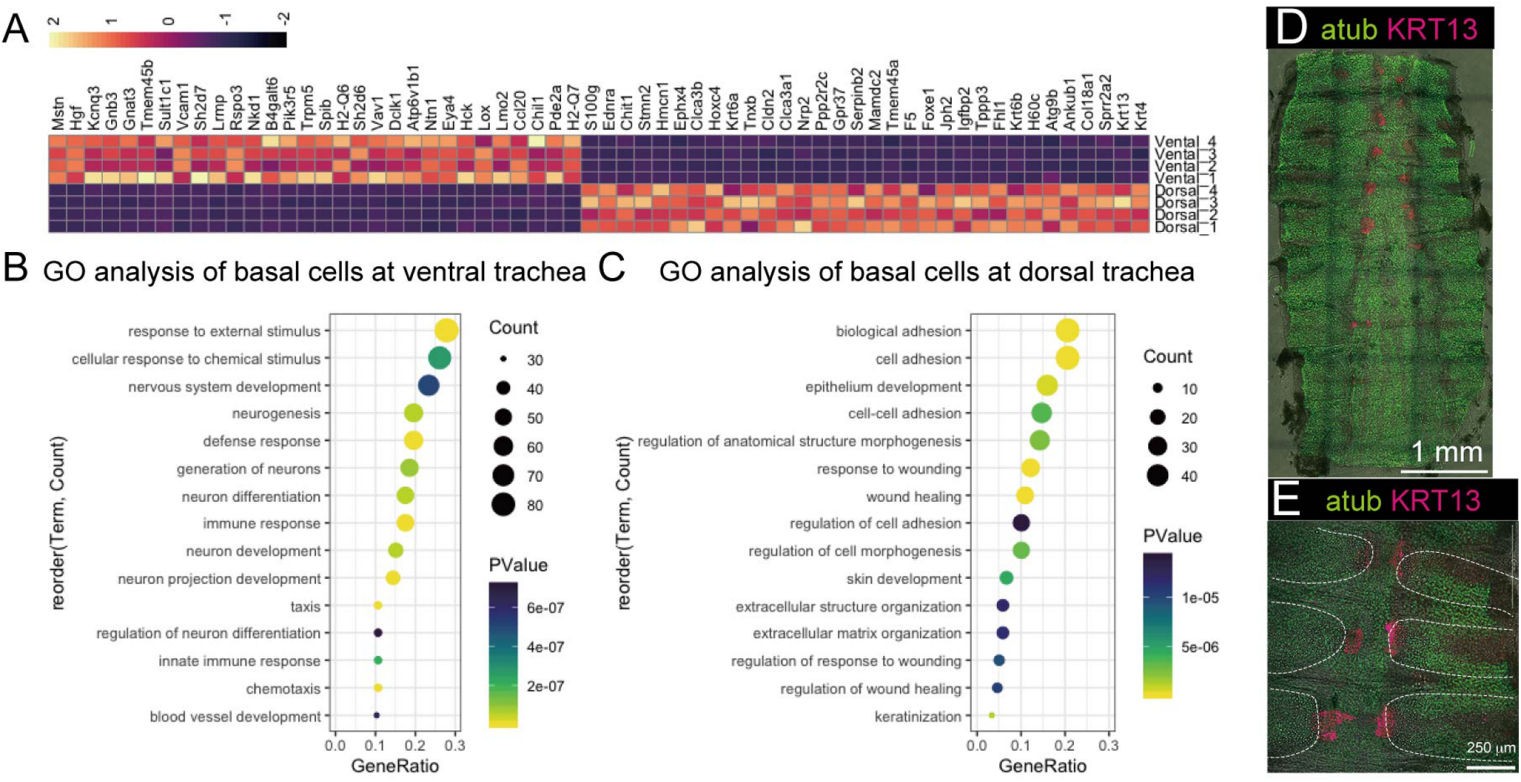
**Comparative gene expression analysis of BCs derived from ventral and dorsal trachea.** (A) Heatmap of enriched genes in BCs derived from ventral and dorsal trachea. (B) GO analysis of enriched genes in BCs derived from ventral trachea using DAVID. (C) GO analysis of enriched genes in BCs derived from dorsal trachea using DAVID. The size of the dot indicates the counts of genes, and the colour indicates the *P*-value (B and C). (D) and (E) Whole-mount immunostaining of dorsal trachea using a ciliated cell marker (atub) and subpopulation of BCs (KRT13). White dotted lines indicate the location of cartilages underneath the airway epithelium.

Since the *in vivo* and *in vitro* proliferation of BCs differed between ventral and dorsal trachea, a gene expression analysis was performed. RNA-seq analysis revealed the top 30 differentially-expressed genes (DEGs) in BCs derived from the ventral and dorsal trachea, which were then indicated in the heat map (Fig. 4A). DEGs (with more than twofold difference in expression levels) were subjected to gene ontology (GO) analysis using DAVID 6.8 (https://david.ncifcrf.gov), a bioinformatics database (Huang et al., 2009a, 2009b). GO analysis revealed that ventral BCs showed enrichment of genes related to immune responses, blood vessel development, and neuron development (Fig. 4B). Among top 30 DEGs, Myostatin, a negative regulator for myogenesis (Thomas et al., 2000), was enriched in ventral BCs. Hepatic growth factor (HGF), which is a hepatic growth factor also known to be involved in chondrogenesis (Wakitani et al., 1997), was enriched in ventral BCs. Although those factors were not involved in the biological processes enriched by GO analysis, those would be involved in BC heterogeneity through sustaining unique microenvironment of ventral trachea. Conversely, the GO analysis revealed that in dorsal BCs, genes related to wound healing processes were enriched. Because the gene expression of KRT6a, KRT13, KRT4, and KRT60b, which are expressed in skin BCs, was also observed in BCs at the anterior part of the dorsal trachea (Fig. 4A,D and E), GO analysis also indicated the enrichment of ‘skin development’ and ‘keratinization’ (Fig. 4C).

## DISCUSSION

In this study, we provide evidence in favour of the heterogeneity in the properties of basal stem cells derived from the dorsal versus ventral regions of mice trachea, which is used as a model system for the conducting airways of the human lung.

Recently, the heterogeneity of BCs has been reported from several research groups along with the rapid progress of scRNA-seq analysis (Watson et al., 2015; Montoro et al., 2018; Braga et al., 2019; Travaglini et al., 2020). By conducting long-term lineage tracing and scRNA-seq analysis, Watson et al. first revealed that there are two subpopulations in BCs: ‘basal stem cells’, and KRT5/KRT8-positive ‘basal luminal cells’, the existence of basal luminal cells is supported by other works (Watson et al., 2015; Mori et al., 2015; Braga et al., 2019; Travaglini et al., 2020). Genes reported to be expressed in basal luminal cells are not changed between ventral and dorsal BCs, suggesting that those two populations are widely distributed in whole trachea. In Watson's work, they analysed BCs only from dorsal trachea, therefore spatial difference of BCs was yet to be addressed.

In our study, some KRT genes are differentially expressed in the dorsal BCs; One is KRT13, which is a squamous cell marker and expressed in BC subpopulation found by single cell RNA-seq analysis (Montoro et al., 2018; Braga et al., 2019). Although Montro et al. mentioned that KRT13-positive BCs are located in the inter-cartilage region, our data suggest that KRT13-positive BCs are present in the dorsal trachea at the boundary between cartilages and smooth muscles, where large BC clones were detected after long-term lineage tracing in our study. This result is also supported by the single cell RNA-sequence analysis of the human respiratory system that BC subpopulation named ‘cycling BCs’ characterised by the high expression of cell proliferation-related genes and KRT13 expression (Braga et al., 2019). Owing to the restricted localisation of KRT13-positive BCs, it is likely that there is a stem cell niche existing between cartilages and smooth muscles. Montoro et al. also suggested that KRT13-positive BCs can only give rise to secretory cells; this finding is consistent with our results, as indicated by the area in the dorsal trachea without FOXJ1 expression while there are covered with ECAD positive luminal cells. Genes expressed in another BC subpopulation called ‘activated basal cells’ found from human lung scRNA-seq analysis (Braga et al., 2019), are not differentially expressed in BCs derived from ventral and dorsal trachea.

Since there were no such differences observed in cell differentiation *in vitro* (data not shown), the differentiation of BCs is considered to be regulated in a non-cell-autonomous manner, specifically, by the mesenchyme lining close to the epithelium. However, careful investigation is required for confirming the differentiation potential of KRT13-positive BCs. Considering their rapid proliferation after injury, BCs, including KRT13-positive BCs in the dorsal trachea, serve as stem cell pools after severe injuries in the tracheal epithelium.

Our results also imply that local BCs play a major role in maintaining homoeostasis in the adjacent mesenchyme. For instance, the expression of myostatin, a known negative regulator of myogenesis (Thomas et al., 2000), in ventral BCs might help maintain an appropriate ratio of smooth muscle mass in the trachea by preventing excess proliferation of smooth muscle cells. To maintain proper ratio of smooth muscle in trachea is thought to be important for normal lung functions because the degree of smooth muscle hypertrophy and hyperplasia in asthma patients is related to disease severity (Bentley and Hershenson, 2008). Besides smooth muscle cell mass, tracheal cartilages are another important structure for respiratory system. Collapse of tracheal cartilage structure observed in tracheomalacia leads to airway block and dyspnea. Our RNA-seq analysis revealed that HGF, which is a growth factor involved in cartilage regeneration (Wakitani et al., 1997), was enriched in ventral BCs compared to dorsal BCs. HGF might help maintain homoeostasis of cartilages in the ventral trachea.

This study revealed the differences in the characteristics of BCs based on spatial distribution, and also indicated the role of epithelial-mesenchymal interactions in tissue homoeostasis in the trachea. The findings of this study suggest that careful observation is necessary in future research, as the influence of environment on the phenomenon should be considered.

## MATERIALS AND METHODS

### Mice

*Krt5*-CreER^T2^ (The Jackson Laboratory), *Rosa26R-CAG-Confetti* (*Rosa-Confetti*; supplied by Hans Clevers)*,* and *Foxj1-*GFP (Ostrowski et al., 2003) mice were maintained on a C57BL/6 background. C57BL/6J mice were purchased from Japan SLC (Shizuoka, Japan). Male mice at 8–12 weeks old were used for SO_2_ injury. All experiments were performed in accordance with Yokohama City University Institutional Guidelines for Laboratory Animal Usage and IACUC-approved protocols at the Duke University School of Medicine.

### 3D culture of tracheospheres

Tracheospheres were cultured according to a method described in a previous report (Rock et al., 2010). In brief, NGFR-positive BCs isolated using fluorescence-activated cell sorting were embedded in growth factor-reduced Matrigel, seeded on cell culture inserts, and cultured for 7 days in MTEC/Plus medium and for 7 days in MTEC/free medium. For sorting, rabbit IgGs were used as a negative control. For quantifying cell growth, tracheospheres isolated from Matrigel by dispase digestion were dissociated using trypsin, and the cells were counted.

### *In vivo* clonal analysis

*Krt5*-CreER^T2^; *Rosa-Confetti* mice (8–12-week-old) were administered tamoxifen in corn oil (10 µg/g body weight) via oral gavage. After 7 days, the mice were treated with SO_2_, and the tracheas were removed at 14 DPI. After fixation with 4% PFA in PBS for 4 h, the tracheas were dissected (ventral and dorsal sections) along the midline. After tissue-clearing treatment using ScaleA2 for O/N (Hama et al., 2011), images of trachea specimens from four mice were obtained using an LSM 710 confocal microscope (Carl Zeiss, Jena, Germany). Cell numbers were determined in clones expressing nuclear GFP in the ventral and dorsal tracheas (71 and 224 clones, respectively). The statistical significance of cell number per clone was determined using the Mann–Whitney–Wilcoxon test.

### Immunohistochemistry

Immunohistochemistry was performed according to a method described in a previous report (Tadokoro et al., 2016). In brief, mouse trachea specimens fixed with 4% PFA in PBS at 4°C for 4 h were washed with PBS and embedded in paraffin. The trachea specimens were sectioned longitudinally along the dorso-ventral axis into 7 µm sections. The sections were deparaffinized, rehydrated, and heated in 10 mM sodium citrate (pH 6.0) at 121°C for 10 min. The sections were blocked by treating with 10% donkey serum, 3% BSA, and 0.1% Triton X-100 in PBS, and were then treated with primary antibodies in blocking buffer at 4°C overnight. The primary antibodies used were as follows: mouse TRP63 (1:50; Santa Cruz Biotechnology, Dallas, TX, USA, SC-8431); rabbit Krt5 (1:500; Covance, PRB-160P), rabbit NGFR (1:100; Abcam, Cambridge, UK, Ab8875), and biotinylated isolectin B4 (1:50; Vector Laboratory, Burlingame, CA, USA). Alexa Fluor-labelled secondary antibodies were used at 1:500 dilution. For detection of GSI-B4 lectin, the sections were stained using Alexa488-labelled streptavidin. The sections were then stained using DAPI and were mounted in FluorSave™ reagent (EMD millipore, San Diego, CA, USA). Images were acquired using an LSM 710 confocal microscope (Carl Zeiss, Jena, Germany).

### Whole-mount imaging of specimens

To visualise ciliated cells, trachea specimens derived from *Foxj1*-GFP mice were fixed in 4% PFA in PBS at 4°C for 4 h. Tiled images were acquired using an LSM 710 confocal microscope (Carl Zeiss, Jena, Germany).

For vascular imaging, the mice euthanised by administering CO_2_ were intracardially perfused with 9.25% (w/v) sucrose followed by perfusion with the fluorescent dye DiI and fixed with 4% PFA in PBS. The images were acquired using the fluorescent microscope Zeiss Axio Zoom.V16 (Carl Zeiss, Jena, Germany).

To visualise BCs, ciliated cells, and nerve fibers, the trachea specimens from C57BL/6J mice fixed with 4% PFA in PBS at 4°C for 4 h were blocked by treating with 10% donkey serum, 3% BSA, and 0.1% Triton X-100 in PBS, and were then treated with primary antibodies (mouse acetylated tubulin antibody, 1:1000, T7451, Sigma-Aldrich, St. Louis, MO, USA; goat Krt13 antibody, 1:500, ab79279, Abcam; mouse β-III tubulin antibody, 1:1000, T8660, Sigma-Aldrich, St. Louis, MO, USA). After subsequent treatment with secondary antibodies (Alexa Fluor488, Alexa Fluor 555), images were acquired using a Leica SP8 confocal microscope and Leica M205 FCA stereomicroscope (Leica Microsystems, Wetzler, Germany).

### cDNA library construction and sequencing

Libraries of each group were prepared from four independent experiments. Total RNA was isolated from NGFR-positive sorted cells using a MoFlo Astrios EQ Cell Sorter (Beckman Coulter, Brea, CA, USA). The quality and quantity of total RNA were evaluated using an Agilent 2100 Bioanalyzer (Agilent Technologies, Santa Clara, CA, USA) and an Agilent RNA 6000 Nano Kit (Agilent Technologies). The cDNA library was constructed using a NEBNext Ultra RNA Library Prep Kit for Illumina (New England Biolabs, Ipswich, MA, USA) according to the manufacturer’ s instructions. Briefly, oligo-(dT) magnetic beads were used to capture poly-(A) mRNA from total RNA, and a fragmentation buffer was added to cleave the mRNAs into short fragments. First-strand cDNAs were synthesized using these short fragments as templates, along with random primers and reverse transcriptase. After second-strand cDNA synthesis, the end-repaired and dA-tailed fragments were connected to sequencing adaptors. The adapter-ligated cDNA fragments were amplified through 11 cycles of PCR, and the products were purified using AMPure XP magnetic beads (Beckman Coulter). The quality and concentration parameters of the library were assessed using the Agilent 2100 Bioanalyzer and an Agilent DNA 1000 Kit (Agilent Technologies). The concentration of the libraries was determined more precisely using a StepOnePlus™ Real-Time PCR System (Applied Biosystems Laboratories, Foster City, CA, USA) and a KAPA Library Quantification Kit (Kapa Biosystems, Wilmington, MA, USA).

The library was diluted to 10 pM and then sequenced using 50-bp single read sequencing with an Illumina HiSeq2500 (Illumina, San Diego, CA, USA). Reads were generated in the FASTQ format using the bcl2fastq2 Conversion Software V2.18.0.12 (Illumina). The read data were submitted to the DDBJ Read Archive (Accession number DRA010271).

### Analysis of DEGs

Gene expression was analysed using the CLC Genomics Workbench 8.5 (Qiagen, Hilden, Germany). First, raw read data were cleaned using the following parameters: quality limit=0.001, ambiguous limit=2, number of 5′ terminal nucleotides=14, number of 3′ terminal nucleotides=3, minimum number of nucleotides in reads=36. The cleaned reads were mapped to GRCm38.p6, the reference genome of *Mus musculus*, retrieved from the Ensembl database (https://asia.ensembl.org/Mus_musculus/Info/Index?db=core). The mapping parameters were as follows: mismatch cost=2, insertion cost=3, deletion cost=3, length fraction=0.8, similarity fraction=0.8. The expression value in each gene was calculated as reads per kilobase of exon per million mapped reads. The number of DEGs was estimated using a statistical approach based on a Gaussian distribution *t*-test. The significant thresholds were set based on fold changes (FCs) of FC≥1.5 or ≤0.67 and a false discovery rate (FDR)-adjusted *P*-value (q<0.05). Genes preferentially expressed in one group (FC≥2) were analysed using DAVID (https://david.ncifcrf.gov/). The results were visualised in the form of a heatmap using R (version 3.5.2) and the pheatmap package on Rstudio (version 1.1.463). For dot plot visualisation, the ggplot2 package was used.

### Statistical analysis

Results are presented as the mean±s.e.m. For tracheosphere assays, three independent experiments were performed in duplicate. For *in vivo* clonal analysis, trachea specimens from four mice were analysed. Statistical significance was determined using unpaired Student's *t*-test, unless otherwise stated.

## Supplementary Material

Supplementary information
