# Supplementary material for: Dorso-ventral heterogeneity in tracheal basal stem cells
Source: Biol Open. 2021 Sep 14;10(9):bio058676. doi: 10.1242/bio.058676 (PMC8467549; doi:10.1242/bio.058676)
Supplement: Supplementary information [file biolopen-10-058676-s1.pdf]

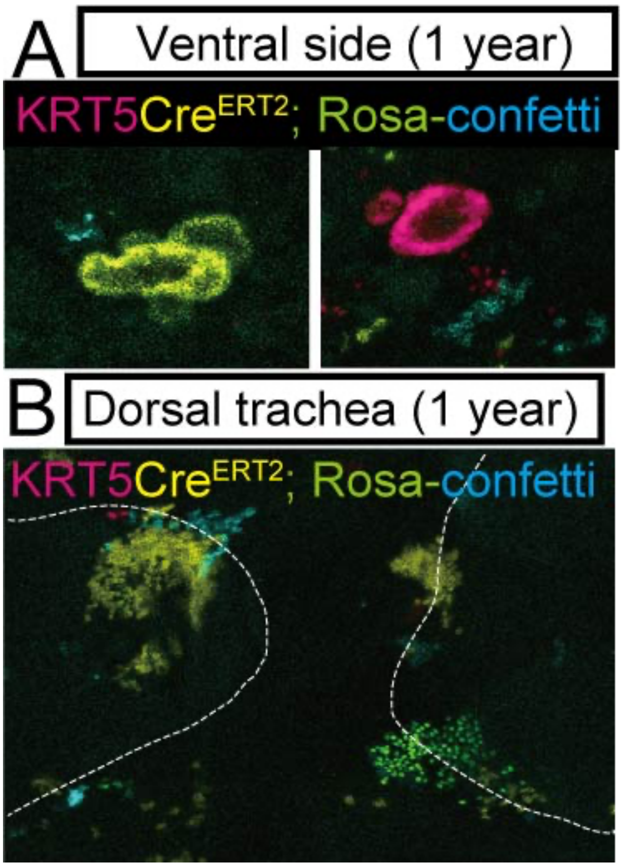

Fig. S1. Whole-mount imaging of long-term lineage tracing of basal stem cells in *Krt5*-Cre^{ERT2}; *Rosa-Confetti* mice. A, Lineage-labelled myoepithelial cell clones from the ventral trachea. B, large clones present in the dorsal trachea. The white dotted lines indicate the locus of cartilages. Related to Fig. 1.

Biology Open • Supplementary information

Table S1. Quantification of cell populations based on marker expression in confocal images in Figure 2G. Related to Figure 2.

	Ventral	Dorsal	P value	Number of specimens
KRT5+TRP63+/KRT5+	88.43 ± 1.07%	89.40 ± 1.31%	0.84651645	15
KRT5+TRP63-/KRT5+	11.57 ± 1.07%	10.60 ± 1.31%	0.84651645	15
Lectin+TRP63+/Lectin+	84.38 ± 1.72%	84.88 ± 1.77%	0.650115213	14
Lectin+TRP63-/Lectin+	15.62 ± 1.72%	15.12 ± 1.77%	0.650115213	14
NGFR+TRP63+/NGFR+	85.16 ± 2.04%	92.12 ± 1.20%	0.008907288	14
NGFR+TRP63-/NGFR+	14.84 ± 2.04 %	7.88 ± 1.20%	0.008907288	14
